# The Effect of COVID-19 Diagnosis on the Physical, Social, and Psychological Well-Being of People in the United Arab Emirates: An Explorative Qualitative Study

**DOI:** 10.3389/fpubh.2022.866078

**Published:** 2022-05-26

**Authors:** Mouza AlKuwaiti, Bayan Abu Hamada, Noof Aljneibi, Marília Silva Paulo, Iffat Elbarazi

**Affiliations:** ^1^Institute of Public Health, College of Medicine and Health Sciences, United Arab Emirates University, Al Ain, United Arab Emirates; ^2^The Emirates Centre for Happiness Research, United Arab Emirates University, Al Ain, United Arab Emirates; ^3^Comprehensive Health Research Center, NOVA Medical School, Universidade Nova de Lisboa, Lisboa, Portugal

**Keywords:** COVID-19, qualitative study, lifestyle changes, physical effects, social impact

## Abstract

A positive COVID-19 infection may impact physical, mental, and social health. Different factors may influence these impacts on different levels due to personal circumstances. This study aimed to explore the impact of a positive COVID-19 diagnosis on the physical,mental, social, psychological health, and lifestyle practices of an individual in the United Arab Emirates. A sample of 28 participants was interviewed using online interviews. An interview guide was created based on the coping strategy model and conceptual framework of coping strategies. All interviews were recorded; then transcribed after obtaining written consent from participants. The NVivo software was used for thematic analysis based on both identified coping models. Major themes included the physical effects, social effects, psychological effects, spiritual effects, and lifestyle effects. Emerging themes include coping mechanisms, trust in authorities and the health care system, appreciation of the role of the government, conspiracy theories, and media roles. This study indicates that people diagnosed with COVID-19 have perceived very good support in terms of their physical health from the government and health authorities, but require social, psychological, and educational support during the infection period and post-recovery.

## Introduction

People around the world were testing positive for SARS-CoV-2; after 1.5 years of the beginning of the pandemic, around 348 million were diagnosed with different COVID-19 variants (1). The world population is seeing unprecedented changes in their daily lives due to the pandemic that affected work, social life, lifestyle, income, and health. These changes that swiped around the world are stressful and cause strong emotions in adults and children (2–5). On top of these stressors, when a person realizes the diagnosis of COVID-19, the increment of all these stress factors and mixed emotions are believed to be exponential for the person as well as their family members and friends. Viruses being unseen, cause psychological distress including fear, denial, and anxiety in the general population (6). A study conducted in Japan demonstrated that close contacts with COVID-19 patients (family and friends) experienced high psychological distress suggesting that the establishment and implementation of mental health and psychosocial support measures tailored to family and close relatives and friends of patients with COVID-19 are warranted (7).

During the past 2 years, so many studies focused on the mental health of the general population and the effect of the pandemic on communities and certain groups (8–13). Studies linked the changes in lifestyle including social isolation, online learning, and strict lockdowns on people's mental, social, and physical health (14, 15). A systematic review found that patients with COVID-19 may suffer from a high level of post-traumatic stress syndrome (PTSD) and a significantly higher level of depressive symptoms, and patients with pre-existing psychiatric disorders reported worsening psychiatric symptoms (5). Among the reported psychological distress caused by COVID-19 are loneliness, anxiety, panic, PTSD, stigma, obsessive behaviors, burnout, hoarding, paranoia, substance abuse and depression, outbursts of racism, and stigmatization. On top of all these reported effects, xenophobia against communities was also being widely highlighted as a result of the COVID-19 pandemic (5).

Since the beginning of the pandemic, research studies have explored mainly the effect of the pandemic on the mental health of general and specific populations. Only a few limited studies have explored the effect of a positive diagnosis on the patient's mental, social, and spiritual effects. Clinical studies reported quantitative short-term and long-term physical effects of the COVID-19 infection on the patients themselves. Therefore, there is a need for studies to explore these effects qualitatively. Moreover, there is also the need of exploring the clinical manifestations during hospitalization, such as a qualitative study that explored the psychological experience of patients with COVID-19 during hospitalization conducted in China. The study by Sun et al. (16) discussed the following five main areas affecting patients with COVID-19: Attitudes toward the disease (fear, denial, and stigma); stress due to the nature of the disease, quarantine measures, and concerns regarding the health of family members; physical and emotional responses, such as lifestyle changes in diet, sleep, and behavior; supportive factors included psychological adjustments, medical care, and family and social support; and finally, gratitude through the cherishing of life, family, bravery, and tenacity. In the United Arab Emirates (UAE), studies have explored similarly the psychosocial impact of the COVID-19 on communities and special populations such as parents, children, university students, and health care professionals (17). However, so far exploring the effect and the impact of the COVID-19 diagnosis on the individual and the experience of the infected person has not been explored through an exploratory qualitative approach in the UAE.

The UAE is considered a relatively young country (50 years old) that emerged from the federation of seven emirates. The country has one of the most competitive health systems in response to its unique population (18). The population is considered unique; although similar to other neighboring countries, as of the 9.7 million people (2019), 87% were expatriates, and there is a bigger proportion of males (69%) compared to females (31%) (19, 20). Another characteristic of the UAE population is the relatively young population, where 84.3% are in the age group of 15–64 years (19, 21, 22).

To provide an overview of the mental, physical, and social effects; and complications of COVID-19 infection among patients in the UAE, it is highly important to explore patients' experiences through a qualitative approach, which allows a better understanding of patients' feelings, experiences, and differences. The study aims to explore the physical, mental, psychosocial, spiritual, and lifestyle impact of a positive COVID-19 diagnosis on UAE residents.

## Methods

This study is being reported according to the consolidated criteria for reporting qualitative research (COREQ) (23). A qualitative approach using online interviews *via* Zoom or Microsoft Teams platforms was utilized. A convenience sample was followed to recruit UAE residents who have had a COVID-19 infection between February 2020 and April 2021. A sample of 28 participants, 25% men and 75% women, of different age groups (23–60) and different nationalities (50% Emiratis) were recruited and interviewed. An interview guide was created based on Lazarus and Folkman's (24) coping strategy model and Burr and Klein's (25) conceptual framework of coping strategies (25).

The definition of coping as described by Lazarus and Folkman (24) is as follows: “constantly changing cognitive and behavioral efforts to manage specific external and internal demands that are appraised as taxing or exceeding the resources of the person” (24). This theory asserts that coping reduces stress by using conscious or unconscious mental solving problems and strategies. These coping strategies can have negative or positive effects on well-being. They depend on personality characteristics and circumstances, and they are never shared. On the contrary, they are very individualized. We hypothesize that people with a positive COVID-19 diagnosis may tend to cope with the social, mental, and physical stress of the infection differently based on their personal experiences, perception of the infection, and social and work pressure considering their economic and political situations. Their coping strategies may vary from physical effects to psychosomatic symptoms to the use of faith and social support.

### Participants' Characteristics and Sample Size

Participants were recruited after the advertisement of the study on WhatsApp through researchers' networks. After selecting the first participant, snowball recruitment was used to identify patients with COVID-19 or those who recovered.

Few participants were known to at least one of the researchers. However, all participants received assurance of confidentiality and privacy. We made sure to assign the known participants to one of the researchers who did not have a previous acquaintance with them.

Inclusion criteria included adult UAE residents, who have had a positive COVID-19 diagnosis and who have recovered between February 2020 and April 2021. Around 50 participants were invited but only 28 agreed to participate. We have tried to have a representation of both genders, different nationalities, and age groups.

### Study Design

We conducted online interviews on Zoom or Microsoft Teams, based on the participants' preferences due to COVID-19 restrictions, social distancing, and “staying at home” campaign. The study used qualitative inductive and deductive approaches. An interview guide was created based on Lazarus and Folkman's (24) coping strategy model and Burr and Klein's (25) conceptual framework of coping strategies. The principal investigator (PI) developed the interview guide in both Arabic and English including questions to explore the mental, social, psychological, and lifestyle impact of a diagnosis of COVID-19. The other researchers reviewed and piloted the interview guide. Among the team there is a researcher who has had COVID-19 at the time and who is also a psychologist and she has added questions related to the social and psychosocial impact and her experience. The interview guide was piloted with five participants who had COVID-19 and who were working in the health care field.

All participants received full information about the study aims, the possibility to withdraw from the research at any time, and confidentiality. Interviews were conducted by trained researchers in qualitative interviews, in both Arabic and English based on the participants' preferences. None of the interviews were repeated.

The participants were approached by phone and *via* WhatsApp. After initial verbal approval by a call to the participant and an information and consent form were sent *via* WhatsApp or email. Upon returning the signed consent form to the researchers, the interview was booked. During the interview, the researchers explained the purpose of the interview, and the privacy and confidentiality procedure. All interviews were video-recorded and then transcribed upon the approval of participants. Most of the interviews were conducted in Arabic, then transcribed and translated. The NVivo qualitative analysis software was used to help in identifying and organizing themes.

Ethics approval was obtained from the UAE University Social Science Ethics Committee (Approval No. ERS_2021_7264).

A research assistant collected all recordings, transcribed them, and translated them from Arabic to English. Then, the researcher imported the English transcriptions to NVivo 12 for analysis and coding. Each interview lasted between 40 and 60 min.

#### Data Saturation

Data saturation was reached after interviewing 15 participants; however, we decided to keep interviewing the participants to ensure we had more representation of the UAE community. Also, to ensure the researchers' reflexivity and to reduce the bias, some of the interviews were transcribed and returned to the participants for review and approval. Participants showed no concerns and were happy with transcribed words.

## Analysis and Findings

### Data Analysis

**Figure 1:**
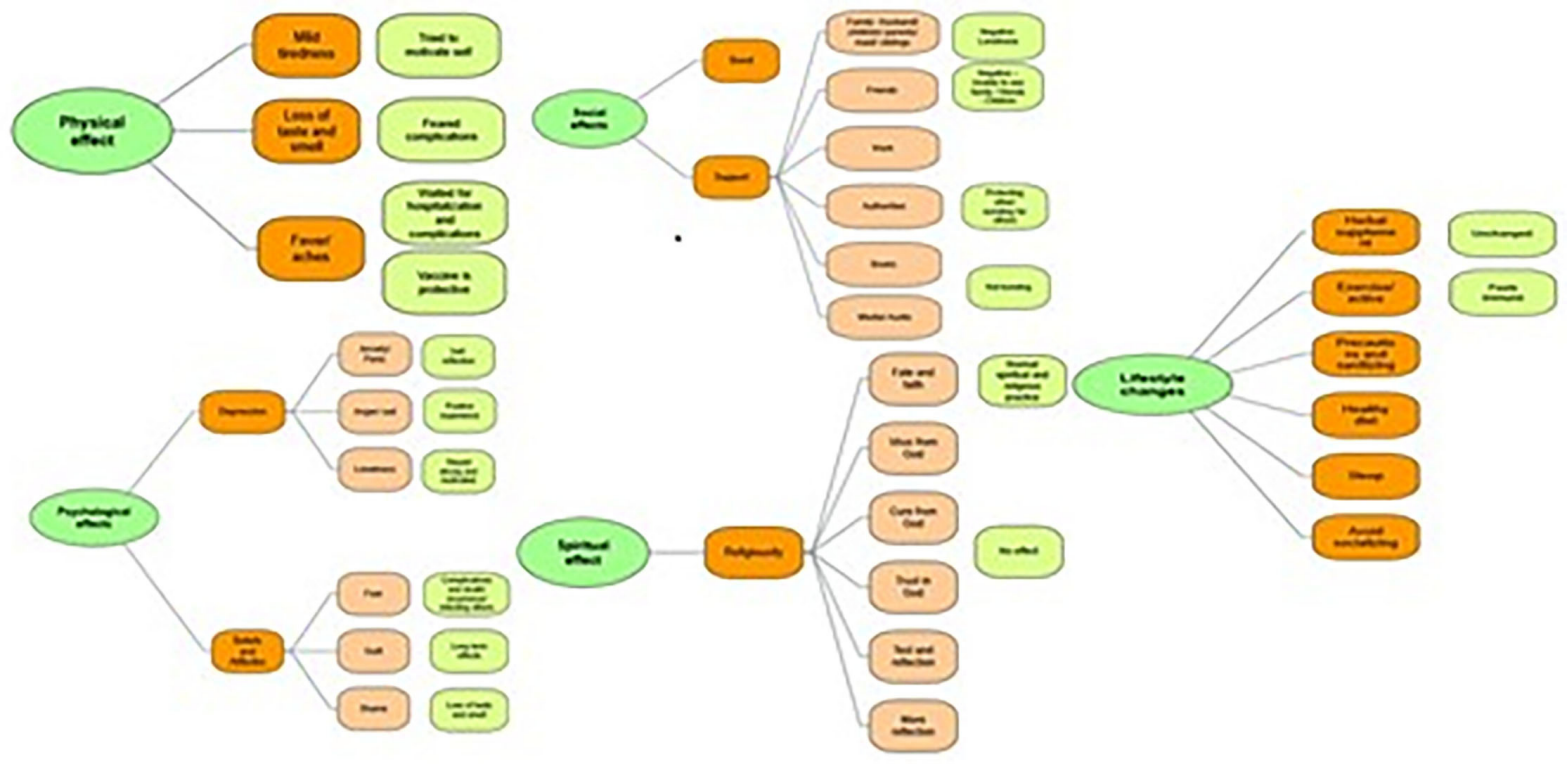
Themes tree^*^. ^*^Physical, social, psychological, physical, and lifestyle impact by NVIVO.

Data analysis was performed using thematic framework analysis as described in Braun and Clarks (2006), six steps process (26). A Thematic tree was created to highlight the most common themes (Figure 1). Authors IE and MSP had revised the transcripts of the interview for triangulation.

As the interview guide was divided into questions related to the physical, social, mental, lifestyle, and spiritual effects of a COVID-19 positive diagnosis on the participant, the coding was based on these five main effects explored. Sub-themes were included under each main major theme.

#### Derivation Themes

Themes derivation was based on the deductive approach from the following reported effects described by the participants: Physical health; social, including the impact of direct family, friends, and social relationships; mental, including the impact on psychological status during and post COVID-19 (returning to normal life); lifestyle changes, including impact on the use of supplements, diet, sleep, and exercise; and spiritual, including faith, fear of death, and their beliefs and spiritual actions.

The derived themes were coded by main categories using NVivo and are presented by category in Figure 1. All effects were divided deductively into the negative and the positive effects of the COVID-19 based on the person's status. Using the inductive approach, other themes were identified that we have classified as emerging themes.

### Results/Findings

Twenty-eight participants were interviewed, half (50%) of them were Emirati nationals, and two-thirds (75%) were women. The demographic characteristics of the participants are presented in Table 1. All participant's codes are presented under the code (participant–number). Major themes include the physical effects, social effects, psychological effects, spiritual effects, and lifestyle effects (see Table 2). Emerging themes include modes of coping and coping mechanisms, trust in authorities and the healthcare system, appreciation of the role of the government, conspiracy theories, and media roles (Table 3).

**Table 1:** Demographic characteristics of the participants.

**Nationality**	**Date of interview**	**Education**	**Age**	**Medical history**	**Job**	**How did they get the virus**	**Date of diagnosis**	**Emirates**	**Vaccine status**
Pakistani	28th_Feb_2021	Doctorate in Clinical Psychology	35	None Disclosed	Clinical psychologist	Hospital	15th_Aug 2021	Dubai	Yes
Emirati	28th_Feb_2021	Bachelor- media	23	None Disclosed	Non	Family- grandma	Sep-20	AD	No
Jordanian	28th_Feb_2021	Bachelor- nutrition	23	None Disclosed	Non	Friend	14-Jan-21	Alain	No
Emirati	2nd_March_2021	Master in Physiology	35	None Disclosed	Director of the happiness center	Not sure	16th_ Jan-2021	Dubai	No
Yemeni	7th_March_2021	High school	58	None Disclosed	Sales	Husband	April-20	Alain	No
Yemeni	10th_March_2021	Master	60	None Disclosed	Non	Work	Apr-20	Alain	Yes
Hungary	11th_March_2021	Doctorate	48	None Disclosed	Professor in IPH	Not mentioned	Jan-2021	Alain	Not mentioned
Egyptian	11th_March_2021	Bachelor's in Vet Medicine	27	None Disclosed	Abu Dhabi Agriculture and Food Safety Authority	Not mentioned	May_2020	Abu Dhabi	Yes
Indian	15th_March_2021	Doctorate	39	None Disclosed	Phd student	Dubai	Jan-2021	Alain	First dose
Lebanese	15th_March_2021	Doctorate	40-50	None Disclosed	Non	Hose helper- not sure	11-Jan-21	Alain	No
Emirati	17th_March_2021	Bachelor	42	None Disclosed	House wife	Don't know	Jan_2021	Alain	Yes
Emirati	21st_March_2021	Bachelor	51	None Disclosed	Physiologist	Husband	Feb_2021	Abu Dhabi	Yes
Emirati	24th_March_2021	Bachelor	22	Asthma patient	Volunteer in AD public health center	Dental clinic	19-May-20	Alain	Yes
Emirati	1st-Aprail_2021	Bachelor	36	None Disclosed	Teacher	Family- husband	March-2021	Alain	No
Emirati	3rd_April_2021	Bachelor	36	None Disclosed	Non	Family- sister-in-law	24-Feb-21	Alain	First dose
Yemeni	28th_April_2021	Master	24	None Disclosed	RA	Friend	Mar-21	Alain	No
Syrian	25_April_2021	Master	40	None Disclosed	Dentist	Don't know	March_2020	Alain	Yes
Emirati	26_April_2021	Master	49	None Disclosed	Manager	Friend	Feb-2021	Alain	Yes
Emirati	26_April_2021	High school	39	None Disclosed	AD police	Not sure	Feb_2021	Alain	Yes
Emirati	27_April_2021	Bachelor	20	None Disclosed	Doctor	Not mentioned	Jan_2021	Alain	Yes
Emirati	6th_May_2021	Bachelor	36	None Disclosed	Teacher	Family	April-2021	Fujairah	Yes
Emirati	6th_May_2021	Bachelor	39	None Disclosed	Banker	Don't know	Oct-2021	Fujairah	Yes
Comoros	6th_May_2021	Bachelor	21	None Disclosed	Student IT	Don't know	Sep_2020	Fujairah	No
Emirati	6th_May_2021	Bachelor	43	Asthma patient	Teacher	Unknown	30-Jul-20	Fujairah	No
Emirati	6th_May_2021	Bachelor	40	None Disclosed	CEO	Family- not sure	Feb-21	Dubai	No
Palestinian	8-May_2021	Bachelor	25	None Disclosed	Costumer service	Unknown	23-Apr-21	AD	First dose
Palestinian	9th_May_2021	Master	25	None Disclosed	RA	Family-not sure	27-Apr-21	AD	First dose
Jordanian	20_June_2021	Master	27	None Disclosed	RA	Family	31_May_21	Alain	Yes

**Table 2:** Major themes emerged.

Major themes^*^	**Sub-Themes -Positive** **impact**	**Sub-Themes-Negative** **impact**
Physical	Acute: Moderate to mild able to cope with	Long term Effects
Social	Bond and support	Loneliness
Psychological	Self-reflection/positive thinking/ appreciation of health	Anxiety/fear/anger/ stigma/shame
Spiritual	Strong relation with God	Unable to perform certain religious activities
Lifestyle effects	Changed lifestyle to healthier die/exercise and sleep and more precautions	Feeling protected/obsessed with precautions

* *Subthemes as extracted and agreed upon by the team researchers*.

**Table 3:** Emerging themes^*^.

**Main themes**	**Sub-Themes**
Coping modes	Self-reflection Time to stop and take a rest Catch up on missed chores Those who contracted COVID-109 at earlier times of the pandemic experienced more fear and anxiety because of uncertainties Blessing of being healthy
Trust in government health authorities	Appreciation of the Effort We will be fine as we have all the support
Conspiracy theories	COVID-19 is man-made, vaccines are part of the conspiracy
Education and media	Media is creating chaos, fear, and anxiety More education is needed Media can be a great tool for education

* *Emerging themes as extracted and agreed upon by the team researchers*.

#### Physical Effects

The majority of the participants (90%) had mild symptoms, from tiredness, fatigue, loss of smell and taste, fever, and body aches. They tried to motivate themselves, thinking that even with their symptoms, they had to stay positive as they believe that their mindset can affect their physical well-being. Some feared more complications, worsening of symptoms, and awaiting hospitalization.

Most of our participants (90%) did not need hospital admission or experienced prolonged serious effects, although 25 participants have mentioned that they did not regain their normal smell or taste sensations. Some stated that the COVID-19 has impacted their general health, and they have not regained their full strength. Three participants referred to some effects such as hair loss and irregular menses. Important to mention, some always fear losing their sense of smell and taste, since it is a distinctive feature of COVID-19. Around eight participants mentioned that they were experiencing a bad smell even after they have recovered. Some participants (*n* = 10) infected in 2021 realized that the vaccine is protective and has helped reduce the severity of the symptoms, especially the ones who took the two doses of the vaccine.

#### Social Effect

The social effect of COVID-19 can be separated into positive and negative effects. The positive effects include bonds and support from family (husband/children/parents/maid/siblings), friends, work, health authorities, books, and media/audio entertainment. The negative effects include loneliness and the restrictions in seeing family, friends, and children. Also it includes feeling of guilt, difficulty to visit and to see parents or to look after them, stigma, stereotype, and feelings of shame and guilt. Protecting others/isolating others and no bonding.

All participants reported receiving a text message of a phone call from health authorities as soon they were diagnosed with a positive infection. Health authorities have shown tremendous efforts in helping and responding to those who turned out positive.

Participant 17 explains as follows:

“*Thank god I got COVID-19 here in the UAE, and not anywhere else, it is a blessing to be in the UAE, everything is vastly available, and the health authorities are just a call away.”*

In the UAE, if a person is infected with COVID-19 with mild–moderate symptoms, they have the option to stay at home in isolation, or in one of the isolation centers. The health authorities also provide food, laundry, and all necessities needed during isolation.

The majority of the participants have received increased support from their family members, husband, children, and co-workers. However, for participants that have no family members in UAE, they have faced some difficulty having social support, nevertheless, with the use of social media, the internet, availability of video calls, they were able to keep in touch with their loved ones.

Some participants (*n* = 5) took time to read books, watch YouTube videos, keep themselves occupied with today's technology, and bond with themselves. One participant mentioned the opportunity to look after her mom while she was admitted to the hospital for COVID-19 as a positive effect. The participant stayed at the hospital to look after her mom and expressed her blessed feeling for being diagnosed with COVID-19 at that time.

#### Psychological Effects

The main negative psychological impact sub-themes extracted included depression, anxiety/panic, anger sad, stigma, guilt, shame, fear of death, fear of complications, and recurrence.

Patients reported shock, anger, and anxiety on the discovery of their positive status. Almost 95% of the participants reported feeling sad, angry, shocked, and scared. Some reported that the anxiety continued throughout their isolation and felt anxious until they received negative testing results and finished the isolation. Their panic was either because of the COVID-19 status or because of the dilemma they faced on how they would break down the news to their families and colleagues from work. This feeling was reported mostly among the ones who got infected at the beginning of the pandemic, where uncertainty and fear of death were at their peak.

Participant 4 and her husband were infected with COVID-19 at the very beginning of the pandemic and she quotes as follows:

“*Fear and anxiety were at their peak at that time, since it was the first time to see a high morbidity, and mortality rates, as well as the information from the media, made it seem like it is the end of the world to get COVID-19”. She also mentions, that her husband was so scared of having COVID-19, that he wrote his “will” because he believed he would die, from what he understood, and saw the start of the pandemic.”*

On a positive note, some participants found that being in isolation was for their benefit. They took some time to self-reflect and take care of themselves, since they understood that we were living in a high-tech era, where most of the people are busy, working, and with social obligations, and during their illness, being forced to take time off made them reflect and do things they did not have time to do before. Some were looking at the bright side, staying strong, and motivated, and that this illness will pass.

Participant 1 referred to this matter by saying the following:

“…*your mental health affects your immunity, and when you have a positive mindset toward the virus and illness, you can boost your immunity, and recover faster.”*

Some people indicated that when they lost their smell and taste senses, they felt the importance of appreciating these abilities by reflecting on what they had and what they lost.

Participant 28 said:

“*When I got the COVID-19 I felt the blessings of tasting food and smelling*.”

##### Beliefs and Attitudes: Fear, Guilt, and Shame

Almost all participants mentioned that they believe the virus is from God, and only God can end this pandemic. Many said, “we have to pray and believe it is happening for our good”. Also, the majority of the participants agreed that the COVID-19 virus is like any other virus, and it will require the contribution of the community and health authorities to fight the virus and end the pandemic. Some participants were afraid of complications that are still unknown about the virus, fear of death, reoccurrence of the disease, and infecting others.

Participant 12 mentions how a strong faith in God is needed in this pandemic, she quotes as follows:

“*Everything is from God, this is all already destined to happen, and we have no control of this, except to trust in God, and do what the authorities tell us to do, of course, we might fear future complications, but we need to keep a strong faith in God, and pray that this will all end.”*

Participant 14 said the following:

“*The recovery period post corona is a new concept among families. People are afraid of corona patients although recovered.”*

The fear of losing jobs is a major issue, considering how businesses have changed since the beginning of the pandemic (e.g., online shopping and delivery of all items). One of the participants has lost his job because due to COVID-19 besides all the regulations imposed by the country to protect the population from stigma.

Fear of transmitting the disease to loved ones has resulted in avoidance of family gatherings, which may have led to feelings of hurt and shame increasing the burden of stigma. Stigma from family members and peers from work has been noticed toward those who are presumed to be affected, are undergoing treatment, or even ones who have recovered from the disease. Nowadays, the term “COVID-positive” has become a stigma, and some might fear telling other family members, colleagues from work, about having COVID-19, due to numerous reasons as follows: Fear of isolation, being far from loved ones, being blamed for the illness, and for being careless.

Participant 9 expressed as follows:

“*Of course, I felt stigma, especially with the people I see regularly, and of course, I understand the paranoia, and how the transmission rates might scare people, but to stigmatize, especially my children, my neighbors would not let her children play with mine in the playground just because I had COVID, even though my kids are negative. There is definitely a stigma, and it does not only affect the person who has the infection, but also the people living with that person can also feel stigmatized.”*

Another participant mentioned how their neighbors refrained from visiting them because of their fear of contracting COVID-19 even after a long period of their recovery and from their negative test results.

#### Spiritual Effects

Most of the participants said that the experience either improved their spirituality and self-reflection or had no influence while none of them stated that the infection and the experience had any negative spiritual or religious effect.

Almost all participants agreed that this is their fate and had reported having strong faith. This statement was shared by almost all participants “The virus is from God, and the cure is from God too. The ones who trust God know that he knows what is best, and it is a test for us.” Many participants reported that they increased their prayers during the illness, while others reported that their faith stayed the same. Keeping a positive mindset and believing all is from God have made it easier for them during isolation. Although for some who were infected during Ramadan, COVID-19 was a barrier to practicing their religious duties.

Participant 18 said the following:

“*During any illness, you do get closer to Allah, and you keep making Dua, because, in the end, you have no one but Allah to talk to about how you are feeling, you know with Quran, Dua, and Praying.”*

Participant 12 said the following:

“I *had more time during isolation to read Quraan on my own, do more prayers at night, and at that time I had the opportunity to be closer to Allah, I had more time to do the things I was behind with my God, I used to finish Surat Al Baqara (a chapter in the Holy Quran) in 1 day, and I feel at that time, really my spirituality level was high at that time, and this gave me more positive power and energy, that I will come out of this stronger than before inshaAllah (God willing).”*

#### Lifestyle Effects

Participants started paying attention to their mental health, spending more time to rest, relaxing during their illness, and keeping a positive mindset. Participants who had more physical symptoms reported that it was difficult to pray, move, and be active in the first few days of their illness. However, they tried their best to walk, move around, and eat better during isolation. After the COVID-19 infection, so many have stated that they returned to their normal lifestyle which they considered to be a healthy one. Few have stated that they decided to follow a healthier lifestyle after their COVID-19 experience to improve their immune system and regain their full health.

Participant 17 mentioned as follows:

“*Ever since COVID happened, I made sure all we buy from the groceries are healthy, organic food, I limited white bread, and I increased their fruits and vegetable intake, to make sure that they have a healthy lifestyle to help them improve their immunity, for the whole family, also limit their time with watching TV, and being active at home.”*

Most participants also mentioned that they were boosting their immune system by having vitamin C, zinc, honey, ginger, and black seed.

Since in UAE, most people believe in the healing properties of herbal supplements, almost all participants have taken herbal supplements, and recipes from families and friends, that claim to relieve their symptoms. Some participants have tried to stay active and exercise even after recovering to help protect them from future illness and reoccurrence.

Some participants who had taken the vaccine and got COVID-19, have reported that they reduced their precautionary activities but were still wearing a mask as recommended with less sanitization while some, reported that they are still sanitizing like at the beginning of the pandemic. While some reported that they are still sanitizing as they did before.

Participant 11 expressed the following:

“*We wear masks because it is mandatory, but I believe when I took the vaccine, and I got COVID-19, I am now more immune, and no need for sanitizing all the time.” While others mentioned, “Since I work in the hospital, I still do all the precautions, even though I am vaccinated, and I got infected with COVID-19, my family and I still sanitize everything, and taking more or so the same precautions as before.”*

A few of the participants have reduced their family gatherings and still avoid socializing with family members, especially the ones who have co-morbidities.

Others stated that they have an unchanged lifestyle as they have had a healthy lifestyle in general before the COVID-19 while others feel immune especially after getting the vaccine and having a positive COVID-19 infection.

Participant 14 said as follows:

“*As you know, my mother and father have many co-morbid conditions, that even my husband told me to wait, we should not be in a hurry and go visit everyone as soon as we are negative, because we are not sure if we can still transmit the disease or not? It is better to be careful than sorry.”*

On another note, participant 11 said as follows:

“*My life has not changed since I recovered from COVID-19, I still visit my family, and have gatherings, I even rewarded myself after I became negative twice to a trip to the Maldives, I am not doing the precautions as before, but I still listen to the authorities, and wear my mask of course.”*

#### Emerging Themes

##### Coping

Participants described utilizing different and various coping mechanisms. Some have tried self-motivation, convincing themselves that their situation is temporary. Others resorted to reading books and the Quran, and watching online videos including motivational and self-help videos, to help cope with the illness.

Participant 12 said the following:

“*Here is where the isolation was a blessing in disguise, for the first time in a very long time, I had time to read my books, watch videos, and documentaries, to entertain myself, it helped me cope with the situation, and I understood that it's just going to be for a while and that I can do all the things I could not do, because of life's high demanding work, family obligations. The isolation helped me, reflect, and do the things I enjoy the most*.”

Five participants (including three working in the medical field) have taken isolation as an opportunity to rest, to self-care, and to catch up with their life. The majority of the participants have used social networking through Zoom, phone, and social media to cope with isolation. Some focused on having a healthy diet, and a good diet to help with their immunity. Finally, religious activities such as praying and reading the Quran were commonly used by most participants.

##### Trust in Government and Health Authorities

One of the very interesting emerging themes was the trust in the healthcare system and the government's role in managing the pandemic. Many of the participants expressed their trust in the treatment protocols and management of the positive cases, indicating that it had a great impact on their journey with the COVID-19 psychologically, physically, and socially. Although some had some negative comments and showed concerns that there was not much support in Dubai such as the one provided in Abu Dhabi; however, overall, many had full trust and appreciation.

##### Conspiracy Theories

When asked about COVID-19 and their experience, around 25% of the participants indicated that COVID-19 is a man-made virus aimed to destroy communities. For others, vaccination was not an option due to their disbelief in the whole situation. Even though some had their doubts, they were still willing to deal with them.

Participant 17 mentioned the following:

“*Even if it is a conspiracy, we have to accept it and face it.”*

For some, doubts existed about the vaccine. A couple of participants expressed their concerns that once they took the vaccine, they became positive.

Participant 18 said as follows:

“*I believe that it was the vaccine that has infected me with COVID-19.”*

##### Education and Media

Finally, it is highly important to mention that the time of infection and the duration played a role on the impact of people psychological and lifestyle. Participants who were diagnosed earlier on during the pandemic had lower knowledge and were receiving confusing messages and information from authorities, health care professionals, and media as the whole situation of COVID-19 was not clear. While those who had the infection at the end of 2020 and in 2021 had better access to clear information which might help them cope better.

Participant 8 said as follows:

“*The last year experience and people stories of recovery make it easier and not scary,”*

Participant 17 also said the following:

“*Number of deaths and uncertainties in the earlier time (2020) was scary.”*

Some have commented on the role of media in exaggerating news and causing chaos calling for more restrictions and actions from authorities.

## Discussion

The study explored the physical, social, psychological, spiritual, and lifestyle effects of a positive COVID-19 diagnosis on a sample of the UAE population. The physical effects reported by the participants varied from loss of smell and taste to tiredness, fatigue, flu-like symptoms, and shortness of breath. Some social effects were reported as positive effects, such as bonding with the family, appreciating family, friends, and neighbors' support. The negative social effects of a COVID-19 infection included stigma of a COVID-19 positive case, discrimination and fear of diagnosed people even after recovery, isolation, fear for loved ones, fear of losing the job, and fear of financial loss. The psychological and mental effects also can be divided into negative and positive. The positive psychological effects of being infected by COVID-19 were feeling blessed and taking time for self-reflection and meditation. The infection served as an opportunity to catch up with missed activities such as reading, watching videos, and connecting with others through social media and long calls. The negative effects were fear, anxiety, anger, and distress and were reported by the majority of participants. The spiritual effects for most participants were positive, as they claimed that the COVID-19 made them closer to God and helped them on improving their spiritual practices. Finally, many participants reported that they were thinking more about a healthier lifestyle to strengthen their immunity and to avoid getting sick again. The participants also mentioned that they would take the vaccine and use vitamins, supplements, honey, and herbal remedies during the period of isolation.

Emerging themes were extracted including different coping strategies, belief in conspiracy, trust in government, and the role of the healthcare system. The study was based on the coping theory which proposes that people cope differently with stressful events based on their cognitive appraisal of the event. The pandemic has created many stressful events in people's individual and social life, and it has created stress on the physical, social, mental, and lifestyle levels. The appraisal of the COVID-19 situation, virus spread, and fear of the unknown may have been most of the factors that have led people to cope differently with these events. Our sample has reported different coping strategies and different ways to adjust to the situations. Some have considered it a positive experience, many have seen it as a very stressful period that may lead to PTSD, as reported by other similar studies. This study reinforces that there is no one-size-fits-all, and it is important to decrease the stress of misinformation and the spread of wrong panicking messages. Moreover, services and hotlines may help improve the way people deal with these stressful situations. Social and community support as well-volunteering services have proven effective to relieve some of these effects and create better coping mechanisms.

The UAE health system has proven to be one of the most efficient during the COVID-19 pandemic with vaccination strategies and mass testing being effective in curbing the spread of the virus and reducing hospitalizations and severe complications (27, 28). However, there is always a need to improve the social and health support for patients with COVID-19 and their close contacts. Many participants reported being very pleased with the health authorities' response to the pandemic in terms of the health services provided (care and attention received). This result was highlighted by residents in Al Ain and Abu Dhabi, under the umbrella of the Department of Health of Abu Dhabi, while residents from the other emirates (not Abu Dhabi) have reported not-so-positive feedback from the support received from their respected health authorities. These differences might be due to the different health authorities and regulatory bodies of each emirate. The UAE health system is unique comprising federal and Emirati-level entities. During the pandemic, a coordinated response from the Ministry of Health and Prevention, the Department of Health of Abu Dhabi, the Abu Dhabi Public Health Center, and the Dubai Health Authority activated a National Technical Advisory Committee to coordinate different health policies and actions across different emirates. The *National Guidelines for Clinical Management and Treatment of COVID-19* were issued in response to global evidence regarding the containment of the virus and treatment of the disease (29), and each regulatory authority issued its guidelines and coordinated the responses within their health systems.

The findings of this study highlight the importance of close support during the infection and after immediate recovery and post-recovery. Social and psychological support is highly needed, especially to deal with the long-term effect of the social and psychological impact of the infection. Serafini et al. (30) reviewed the literature and found that resilience, loneliness, anxiety, loss of freedom, and well-being were the main psychological effects of COVID-19 on communities (30). This may typically increase with being affected directly or having a loved one affected by the infection. The same authors and others discussed the impact of the COVID-19 pandemic on lifestyle and social isolation (30–33). The study participants reported many changes in their lifestyle during the infection, especially isolation, loss of job, and being away from others but other changes may last after the infection such as the increase in the use of precautions measures (handwashing, sanitizers, changes in diet, and other lifestyle factors). Proper advice and education are mandatory in these cases, one of the participants was a psychologist who pointed out the increase in obsessive–compulsive behaviors among people due to the fear of infection. This was also questioned by Jelinek et al. (34).

During the interviews, we noted that beliefs toward the virus have changed from people who got infected in 2020 compared to those who got infected in 2021. In 2021, people's knowledge and acceptance of the vaccination, fear of dying from COVID-19, and the unknown long-term and short-term effects had decreased with time and information. Studies assessing knowledge and awareness about COVID-19 and its vaccines have shown that positive attitudes have increased due to the local governments' efforts and global education (35). The study shows that there are common coping strategies with a positive COVID-19 diagnosis. However, there are common issues shared that may need to be addressed to improve the coping mechanism for this population and their families.

A significant finding in this study was that those who had a positive infection toward the end of 2020 and in 2021 described having fewer negative emotions and better psychological resilience. They referred to the fact that they had better knowledge about the COVID-19 effects, availability of the vaccines, and governments' efforts to ease the pandemic. Although some participants expressed their satisfaction with the support they received, a large number of participants (almost 90%) reported their strong appreciation for the UAE government, and health authorities reflected on satisfactory knowledge and favorable practice with an overall high positive attitude (35).

The COVID-19 hotline provided by the health authorities identified people who may be infected but did not focus on providing psychological support to help with the patients' fears and doubts. Although there is an improvement in knowledge, awareness and self-copying strategies are still critical in this pandemic.

It is crucial to educate newly diagnosed patients about COVID-19, and the most recent knowledge about it, to prevent people from being overwhelmed with stigma from their society and the media. Another important point is to inform patients diagnosed with COVID-19 to report any mental or social issues that can be solved during the course of their physical symptoms. Most of these patients have had all the support they needed, but we cannot deny that there is a need to focus more on the mental and social well-being of these patients because they understand that their immune system is affected by their mental health. The participants have perceived very good support in terms of their physical health from the government and health authorities. Finally, the study shows that there are common coping strategies for a positive COVID-19 diagnosis. However, there are common issues shared that may need to be addressed to improve the coping mechanism for this population and their families.

### Limitations

This is a qualitative study, and we cannot generalize the results to the general population. However, being an explorative study, further studies can build on our findings. Another limitation of this study is the COVID-19 situation, where lockdowns and the chaos caused in the society limited our access to participants and to conduct face-to-face interviews. Moreover, many participants approached were reluctant to discuss their experiences.

## Conclusion

The findings of this study indicate that people diagnosed with COVID-19 have perceived very good support in terms of their physical health from the government and health authorities, but require social, psychological, and educational support during the infection period and post-recovery. This support may be needed for a longer period to prevent further implications on individuals' and families' physical and psychological health.

## Data Availability Statement

The original contributions presented in the study are included in the article/Supplementary Material, further inquiries can be directed to the corresponding author.

## Ethics Statement

Ethical approval was obtained from the United Arab Emirates University Social Sciences Ethics Committee (Approval No. ERS_2021_7264).

## Author Contributions

IE conceptualized the study. IE, BH, MA, and NA conducted the interviews and analyzed the data. IE, BH, and MA agreed on the themes. IE, MA, MSP, and NA wrote and reviewed the manuscript. All authors accepted the manuscript for publication.

## Conflict of Interest

The authors declare that the research was conducted in the absence of any commercial or financial relationships that could be construed as a potential conflict of interest.

## Publisher's Note

All claims expressed in this article are solely those of the authors and do not necessarily represent those of their affiliated organizations, or those of the publisher, the editors and the reviewers. Any product that may be evaluated in this article, or claim that may be made by its manufacturer, is not guaranteed or endorsed by the publisher.

## Abbreviations

COREQ: Consolidated criteria for reporting qualitative research
COVID-19: Coronavirus disease 2019; Master of Public Health; Post-traumatic stress symptoms
UAE: United Arab Emirates.
