# Supplementary material for: The Effect of COVID-19 Diagnosis on the Physical, Social, and Psychological Well-Being of People in the United Arab Emirates: An Explorative Qualitative Study
Source: Front Public Health. 2022 May 26;10:866078. doi: 10.3389/fpubh.2022.866078 (PMC9199957; doi:10.3389/fpubh.2022.866078)
Supplement: Supplementary file 1 [file Data_Sheet_1.PDF]

Effects of COVID-9 infection on person's mental health, lifestyle practices, family relations and social connectedness

Interview Guide

A. Demographics

B. Questions on events:

- What do you think of the COVID-19 infection? Beliefs and attitudes toward the virus (origin of the virus, causes, severity, long term and short term effects)
- How would describe your condition during the COVID-19 Infection?

C. Coping behaviour

- External resources: How did you deal with the infection (Medical support, pharmacological support) DOH/MOHAP/DOHA support-) Home care support, testing and advice?
- Internal resources: Family and friends support, workplace and colleagues support- Check the stigma, feeling of guilt , shame and fear to infect close ones- How did it affect on your relationships with others

D. Psychological consequences:

- How did the infection affect your mental health? (ask about anger, anxiety, fear of death, fear of complications, anger, sadness, worry, numbness, or frustration, worsening of chronic disease, mental health, panic attacks.
- Did you seek mental health help? Ask about coping mechanism (Did you speak with a specialist: counselor, friend , family and friends about your feeling?) Did you attend any courses? Any special youtube videos etc…) Did you sleep more, changed your lifestyle to cope??

E. Physical Consequences:

- What are the physical symptoms you developed? Any headache (Did you suffer from headaches before?) Any increased heart rate, body ache? How did this affect you? What about loss of taste and smell? Did it subside? Describe your feelings about the loss of taste and smell? Any associated fears, anxiety….

F. Social consequences including work

- How did the infection affect your family relationships/ friends relationship/ work impact and colleagues' relationships? Describe in details (prompt did it create bonding, problems, anger,… What about your extended family ?

G. Lifestyle changes (Diet, physical activity, smoking, sleeping, use of internet, studies, work, religiosity, mind exercise)

- Ask if the covid19 positive status affected their lifestyle use components above during and after the infection find about in details what happened and why they changed if it happened

H. Future implication of the virus on you (fear of complications, recurrent infections, vaccine)

- Ask if the person has any concerns about any future physical, mental social complications due to COVID-19 infection- if they are stronger fear of recurrent infection and possibility to cope and vaccine willingness to take the vaccine or feeling that he/she is immune and no need to vaccinate at least this year.
